# TLR2 and the NLRP3 inflammasome mediate IL-1β production in *Prevotella nigrescens*-infected dendritic cells

**DOI:** 10.7150/ijms.47197

**Published:** 2021-01-01

**Authors:** Hye-Mi Jang, Ji-Yeon Park, Yeon-Ji Lee, Min-Jung Kang, Sung-Gang Jo, Yu-Jin Jeong, Nam-Pyo Cho, Sung-Dae Cho, Dong-Jae Kim, Jong-Hwan Park

**Affiliations:** 1 Laboratory Animal Medicine, College of Veterinary Medicine and BK 21 PLUS Project Team, Chonnam National University, Gwangju, Republic of Korea.; 2 Department of Oral Pathology, School of Dentistry, Institute of Biodegradable material, Institute of Oral Bioscience, Chonbuk National University, Jeonju, Republic of Korea.; 3 Infectious disease Research Center, Korea research institute of bioscience & biotechnology, Daejeon, Republic of Korea.; 4 Department of Oral Pathology, School of Dentistry and Dental Research Institute, Seoul National University, Seou, Republic of Korea.; 5 Laboraotry Animal Resource Center, DGIST, Daegu, Republic of Korea.

**Keywords:** *Prevotella nigrescens*, IL-1β, TLR2, NLRP3 inflammasome, BMDCs

## Abstract

*Prevotella nigrescens* is an oral pathogen that is frequently observed in the subgingival plaque of periodontitis patients. Interleukin-1β (IL-1β) is known to be involved in the immunopathology of periodontal diseases and has been implicated in the destruction of bone. In this study, we investigated the mechanism of IL-1β production by* P. nigrescens* in murine bone marrow-derived dendritic cells (BMDCs). Our results showed that a host receptor, Toll-like receptor 2 (TLR2), but not TLR4 is required for pro-IL-1β induction and nucleotide-binding oligomerization domain like receptor pyrin domain containing 3 (NLRP3) priming in BMDCs in response to *P. nigrescens* and activation of the NLRP3 inflammasome is necessary for processing of pro-IL-1β into mature IL-1β. In addition, an inhibitor assay revealed that production of reactive oxygen species, P2X_7_R activity, and release of cathepsin B are involved in IL-1β production in BMDCs in response to *P. nigrescens.*

## Introduction

Periodontitis is a chronic inflammatory disease characterized by alveolar bone resorption; it is the major cause of tooth loss in adults 1-4. Moreover, periodontitis is associated with systemic diseases such as atherosclerosis, Type 2 diabetes, rheumatoid arthritis, and chronic obstructive pulmonary disease 5-7. The periodontal tissue is constantly exposed to oral pathogens, such as *Porphyromonas gingivalis*, *Fusobacterium nucleatum,* and *Prevotella nigrescens,* and other black-pigmented gram-negative anaerobes in the bacterial plaque that are known to induce periodontitis 1,2,8,9.

Interleukin-1β (IL-1β) is an important pro-inflammatory cytokine involved in many cellular functions, such as proliferation, differentiation, and activation of immune cells. It plays a critical role in the innate immune response 4,10. It also controls initiation and progression of periodontal disease by directly stimulating bone destruction and triggering the release of prostaglandin E2 and matrix metalloproteinases (MMPs), which activate immune cells and inhibit collagen synthesis, respectively 4. The production of IL-1β is tightly regulated in two steps. The first step is transcriptional induction of pro-IL-1β (precursor of IL-1β), which is stimulated by recognition of microbial structures by pattern recognition receptors (PRRs) such as Toll-like receptors (TLRs), nucleotide-binding oligomerization domain-like receptors (NLRs), and retinoic acid-inducible gene (RIG)-like helicases. The second step is enzymatic cleavage of pro-IL-1β into mature IL-1β by proteases including caspase-1 3,11. Activation of caspase-1 is mediated by the inflammasome, which is an intracellular multiprotein complex consisting of an upstream NOD-like receptor family, the adaptor protein apoptosis-associated speck like protein containing a caspase recruitment domain (ASC), and the downstream effector caspase-1 12. To date, several NLR proteins, including NLR pyrin domain-containing 3 (NLRP3) and NLR caspase domain-containing 4 (NLRC4), as well as absent in melanoma 2 (AIM2) have been reported to initiate the formation of an inflammasome 8. Upon stimulation with pathogen-associated molecular patterns (PAMPs) or damage-associated molecular patterns (DAMPs), inflammasome assembly leads to the autocatalytic cleavage of caspase-1 and processing of pro-IL-1β into mature forms 1,12,13. Activation of NLRP3 can be induced by ATP, bacterial pore-forming toxins, potassium efflux, generation of mitochondrial reactive oxygen species (ROS), and cathepsin B release by lysosomal destabilization. NLRC4 can be activated by cytosolic bacterial flagellin and rod and needle components of the type III secretion system 13.

Dendritic cells (DCs) play a critical role in the initiation and progression of both innate and adaptive immunity. As a sentinel in peripheral tissues, DCs capture and process pathogens and recognize PAMPs using PRRs. As a result, DCs express C-C chemokine receptor type 7 (CCR7) and migrate to the lymph nodes (LNs). During migration, DCs up-regulate the expression of MHC class II and co-stimulatory molecules which can present antigens to CD4^+^ T cells. Antigen-recognized CD4^+^ T cells leave the LNs and enter circulation, infiltrate the infected tissue, and exert their immune effector functions. DCs rapidly monitor the accumulation of bacterial dental plaque in the supra- and subgingival regions and induce T cell-mediated immune responses. In addition, DCs produce several pro-inflammatory cytokines, including IL-1β, which is known to be involved in immunopathology of periodontal diseases and has been implicated in the destruction of bone 4.

*P. nigrescens*, an endodontic pathogen that belongs to the *Bacteroidaceae* family, is frequently observed in the subgingival plaque of periodontitis patients 8,14. In the present study, we sought to determine the molecular mechanism of host IL-1β production following *P. nigrescens* infection using BMDCs; we show that TLR2 is necessary to induce expression of pro-IL-1β and NLRP3 and that the NLRP3 inflammasome is required to process immature IL-1β into mature IL-1β.

## Materials and Methods

### Mice

TLR2^-/-^ and TLR4^-/-^ mice on a C57BL/6 background were purchased from the Jackson Laboratories (Bar Harbor, ME, USA). NLRP3^-/-^, NLRC4^-/-^, Caspase-1/11^-/-^, and ASC^-/-^ mice in a C57BL/6 background have previously been described 15. Wild-type (WT) C57BL/6 mice were purchased from Koatech (Pyeongtaek, South Korea). Protocols for animal studies were approved by the Institutional Animal Care and Use Committee of Chonnam National University (Gwangju, Korea).

### Bacterial preparation

*P. nigrescens* strain KCTC 15081 (ATCC 33563) was purchased from the Korean Collection for Type Cultures (Jeongeup, South Korea). Stock broths were inoculated into 10 mL of brain heart infusion (BHI) broth with hemin (5 mg/mL) and menadione (10 mg/mL) under anaerobic conditions at 37°C in an incubator. A 1:10 dilution of the overnight culture was prepared and allowed to grow with shaking to an optical density (at a wavelength of 600 nm) of 0.6, which corresponds to ~10^9^ colony-forming units (CFU)/mL of viable bacteria by serial dilution and plate counts. After washing with phosphate-buffered saline (PBS; pH 7.4), bacteria was diluted to the desired concentration with PBS. The bacteria were then exposed to heat (95°C for 15 min).

### Preparation of BMDCs and infection with *P. nigrescens*

Murine bone marrow-derived dendritic cells (BMDCs) were prepared as previously described 16. Briefly, BMDCs were cultured with RPMI media containing GM-CSF (20 ng/mL), 1% penicillin/streptomycin, 1% L-glutamine, 10% FBS, and 2-mercaptoethanol (0.1 μg/mL) in a 5% CO_2_ incubator at 37°C, and fresh media was added on days 3 and 6. After 9 days, non-adherent cells were collected by vigorous aspiration. For cytokine measurement, the cells were seeded in 48-well plates at a concentration of 2×10^5^ cells/well and incubated in a 5% CO_2_ incubator at 37°C for 2 h. Subsequently, the cells were infected with *P. nigrescens* at the indicated multiplicity of infection (MOI) (uninfected cells were used as controls). Culture supernatant was collected 24 h after infection for cytokine measurement.

### Measurement of cytokines

The concentrations of IL-6 and IL-1β in the culture supernatants were determined using a commercial enzyme-linked immunosorbent assay (ELISA) kit according to the manufacturer's protocol (R&D systems, Minneapolis, MN, USA). The absorbance was measured using a microplate spectrophotometer (BioTek, Winooski, VT, USA) at 450 nm.

### Immunoblotting

For immunoblotting, BMDCs from WT, caspase-1-, ASC-, NLRP3-, and NLRC4-deficient mice were seeded and incubated for 2 h in 6-well plates at a concentration of 2×10^6^ cells/well and then infected with *P. nigrescens* at MOI 1, 10, and 100. 6 or 24 hours after infection, BMDCs were lysed with or without culture supernatant by adding 1% Nonidet P-40 (Sigma), complete protease inhibitor cocktail (Roche), and 2 mM dithiothreitol. Samples were mixed with SDS buffer, boiled for 10 min, separated by SDS-PAGE, and transferred onto nitrocellulose membranes. The membranes were incubated with the following primary antibodies: anti-mouse caspase-1 (Enzo Life Science, Farmingdale, NY, USA), anti-mouse IL-1β (R&D Systems), anti-P2X_7_ Receptor (Sigma, St. Louis, MO, USA), anti-Cathepsin B (abcam, Cambridge, MA, USA) and anti-β-actin (Santa Cruz biotechnology, Santa Cruz, CA, USA). A primary antibody against anti-β-actin was used to verify equal loading of protein samples. Following incubation, the relevant secondary antibodies (Santa Cruz Biotechnology, Dallas, TX, USA) were used and proteins were detected with chemiluminescence (ECL) reagent (BioRad, Hercules, CA, USA) and visualized with an Amersham Imager 600 (GE Healthcare Bio-sciences AB, Sweden). To prepare positive control samples for caspase-1 activation and IL-1β production, cells were treated with LPS (100 ng/mL) for 6 h with or without additional ATP (2 mM) treatment for 40 min.

### cDNA synthesis and Real-time PCR

Gene expression levels of IL-1β and NLRP3 were determined by real-time PCR. BMDCs from WT and TLR2-deficient mice were infected with *P. nigrescens* at an MOI of 100 for 2 h and RNA was extracted using the easy-BLUE^TM^ Total RNA Extraction Kit (iNtRON Biotechnology, Seongnam, Korea). cDNA was synthesized from 0.1 μg of RNA using ReverTra Ace® qPCR RT Master Mix (TOYOBO Bio-Technology, Osaka, Japan) according to the manufacturer's instructions. PCR was performed using the QGreen^TM^ 2X SybrGreen qPCR kit (Qiagen GmbH, Hilden, Germany). GAPDH was used for normalization of expression levels. The following primer sequences were used: IL-1β (IL-1β forward 5'-GATCCACACTCTCCAGCTGCA-3', IL-1β reverse 5'-CAACCAACAAGTGATATTCTCCATG-3'); NLRP3 (NLRP3 forward 5'-ATGGTATGCCAGGAGGACAG-3', NLRP3 reverse 5'-ATGCTCCTTGACCAGTTGGA-3'); AIM2 (AIM2 forward 5'-AACCCAAGCAAAACAAAGTG-3', AIM2 reverse 5'-GCTACAAGGTCCAGATTTCAAC-3'); ASC (ASC forward 5'-TCACAGAAGTGGACGGAGTG-3', ASC reverse 5'-TCATCTTGTCTTGGCTGGTG-3'); Caspase-1 (Caspase-1 forward 5'-GCCCACTGCTGATAGGGTGA-3', Caspase-1 reverse 5'-CCCGGGAAGAGGTAGAAACG-3'); NLRC4 (NLRC4 forward 5'-ACGCAGGCAAAACACTCATA-3', NLRC4 reverse 5'-TCGTTTCTCAAGCCAATTCC-3'); and GAPDH (GAPDH forward 5'-CGACTTCAACAGCAACTCCCACTCTTCC-3', GAPDH reverse 5'-TGGGTGGTCCAGGGTTTCTTACTCCTT-3'). PCR amplification was performed using a three-step protocol of 95°C for 10 seconds followed by 40 cycles of 58°C for 15 seconds, and 72°C for 20 seconds in a Rotor-Gene Q real-time PCR system (Qiagen).

### DCFDA assay

ROS production was measured by staining with 2',7'-dichlorofluorescin diacetate (DCFDA, Sigma) according to the manufacturer's instructions. In brief, BMDCs from WT mice were infected with *P. nigrescens* at MOI 1, 10, and 100 for 24 h. The cells were washed twice with PBS, and then incubated with 15 μM DCFDA for 10 min at 37°C. Subsequently, the cells were washed with PBS and detected using a fluorescence microscope.

### Inhibitor assay

N-Acetyl-L-cysteine (NAC), oxidized ATP (oxATP), and Glyburide were purchased from Sigma-Aldrich (St. Louis, MO, USA). CA-074 methyl ester (CA-074 Me) and Ac-YVAD-CMK (Y-VAD) were purchased from Calbiochem (La Jolla, CA, USA). BMDCs from WT mice were infected with *P. nigrescens* at an MOI of 10 with or without pretreatment with NAC, oxATP, CA-074 Me, Glyburide, or Y-VAD for 2 h. Twenty-four hours after infection, the concentrations of IL-1β and IL-6 in the culture supernatant were measured by ELISA.

### Statistical analysis

Statistically significant differences between groups were determined with the two-tailed Student's *t*-test or one-way analysis of variance (ANOVA) followed by *post hoc* analysis (Newman-Keuls multiple comparison test) (Graphpad Prism version 5). Values of *P < 0.05 were considered statistically significant.

## Results

### BMDCs produce IL-1β and IL-6 in response to *P. nigrescens* infection

**Figure 1:**
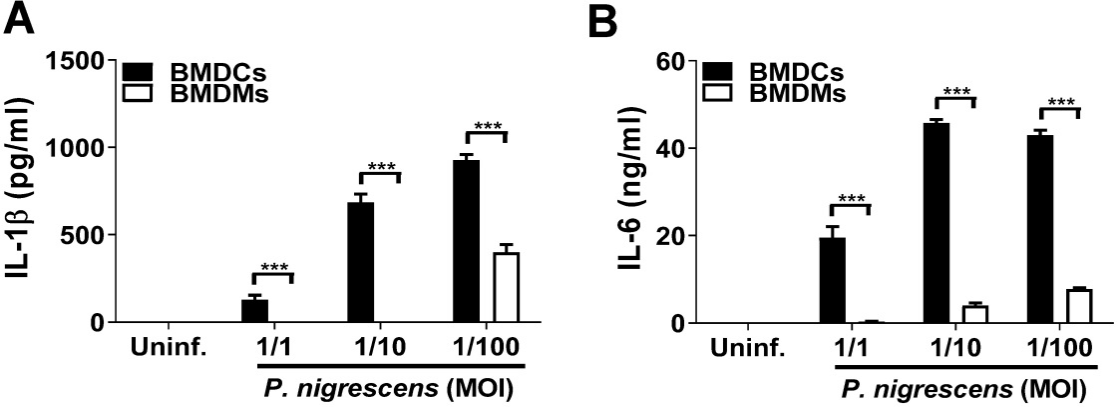
** BMDCs produce IL-1β and IL-6 in response to *P. nigrescens* infection.** BMDMs and BMDCs from WT mice were infected with *P. nigrescens* at the indicated MOI*.* Twenty-four hours after infection, culture supernatants were collected and (A) IL-1β and (B) IL-6 production was measured by ELISA. The results shown are from one experiment representative of three independent experiments and expressed as mean ± SD. *P < 0.05, **P < 0.01, and ***P < 0.001.

Dendritic cells (DCs) in the oral mucosa play a pivotal role in periodontitis 6. Although murine macrophages have been shown to produce IL-1β and IL-6 in response to *P. intermedia* LPS treatment 17, the immune response by DCs to *Prevotella* infection remains poorly understood. To determine the immune response of DCs to *P. nigrescens* infection, BMDCs were infected with *P. nigrescens*. BMDCs produced IL-1β and IL-6 in response to *P. nigrescens* infection in a dose-dependent manner (Fig. 1A and B). Moreover, the level of cytokines produced following infection was higher in BMDCs than in BMDMs (Fig. 1A and B). These results indicate that BMDCs produce IL-1β and IL-6 in response to *P. nigrescens* infection.

### TLR2, but not TLR4, contributes to production of IL-1β and IL-6 and mRNA expression of IL-1β and NLRP3 in *P. nigrescens*-infected BMDCs

**Figure 2:**
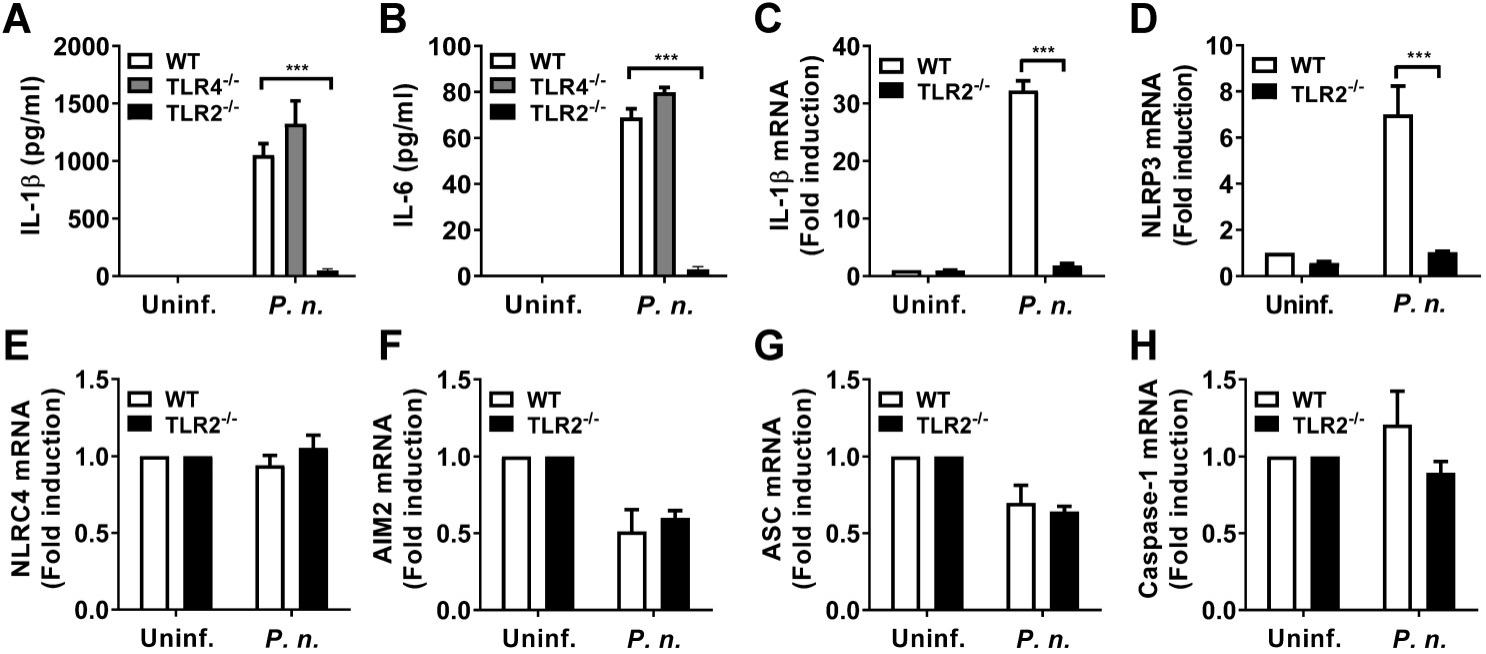
** TLR2, but not TLR4, contributes to production of IL-1β and IL-6 and mRNA expression of IL-1β and NLRP3 in *P. nigrescens*-infected BMDCs.** BMDCs from WT, TLR2-, and TLR4-deficient mice were infected with *P. nigrescens* at an MOI of 10 for 24 h. After infection, culture supernatant was collected and (A) IL-1β and (B) IL-6 production was measured by ELISA. BMDCs from WT and TLR2-deficient mice were infected with *P. nigrescens* at an MOI of 100 for 2 h and mRNA expression of (C) IL-1β, (D) NLRP3, (E) NLRC4, (F) AIM2, (G) ASC, and (H) caspase-1 was evaluated by real-time PCR. The results shown are from one experiment representative of three independent experiments and expressed as mean ± SD. *P < 0.05, **P < 0.01, and ***P < 0.001.

A previous study showed that gram-negative periodontal bacteria stimulated host TLR2 and TLR4 18. To identify host factors required for production of IL-1β and IL-6 in *P. nigrescens*-infected cells, BMDCs from WT, TLR2-, and TLR4-deficient mice were infected with *P. nigrescens*. The production of IL-1β and IL-6 in BMDCs from TLR4-deficient mice was comparable with that in BMDCs from WT mice (Fig. 2A and B). However, BMDCs from TLR2-deficient mice showed impaired production of IL-1β and IL-6 compared to those from WT mice (Fig. 2A and B). Transcriptional induction of pro-IL-1β and NLRP3 is required for IL-1β processing by the NLRP3 inflammasome 12. BMDCs from TLR2-deficient mice showed impaired transcriptional induction of IL-1β and NLRP3 compared to those from WT mice in response to* P. nigrescens* infection (Fig. 2C and D). Expression of other genes related to inflammasome signaling such as NLRC4, AIM2, ASC, and caspase-1 was comparable between WT and TLR2-deficient BMDCs (Fig. 2E-H). These results indicate that TLR2, but not TLR4 contributes to production of IL-1β and IL-6 and transcriptional induction of IL-1β and NLRP3 in BMDCs in response to* P. nigrescens* infection.

### Caspase-1 and ASC are required for *P. nigrescens*-induced processing of IL-1β in BMDCs

**Figure 3:**
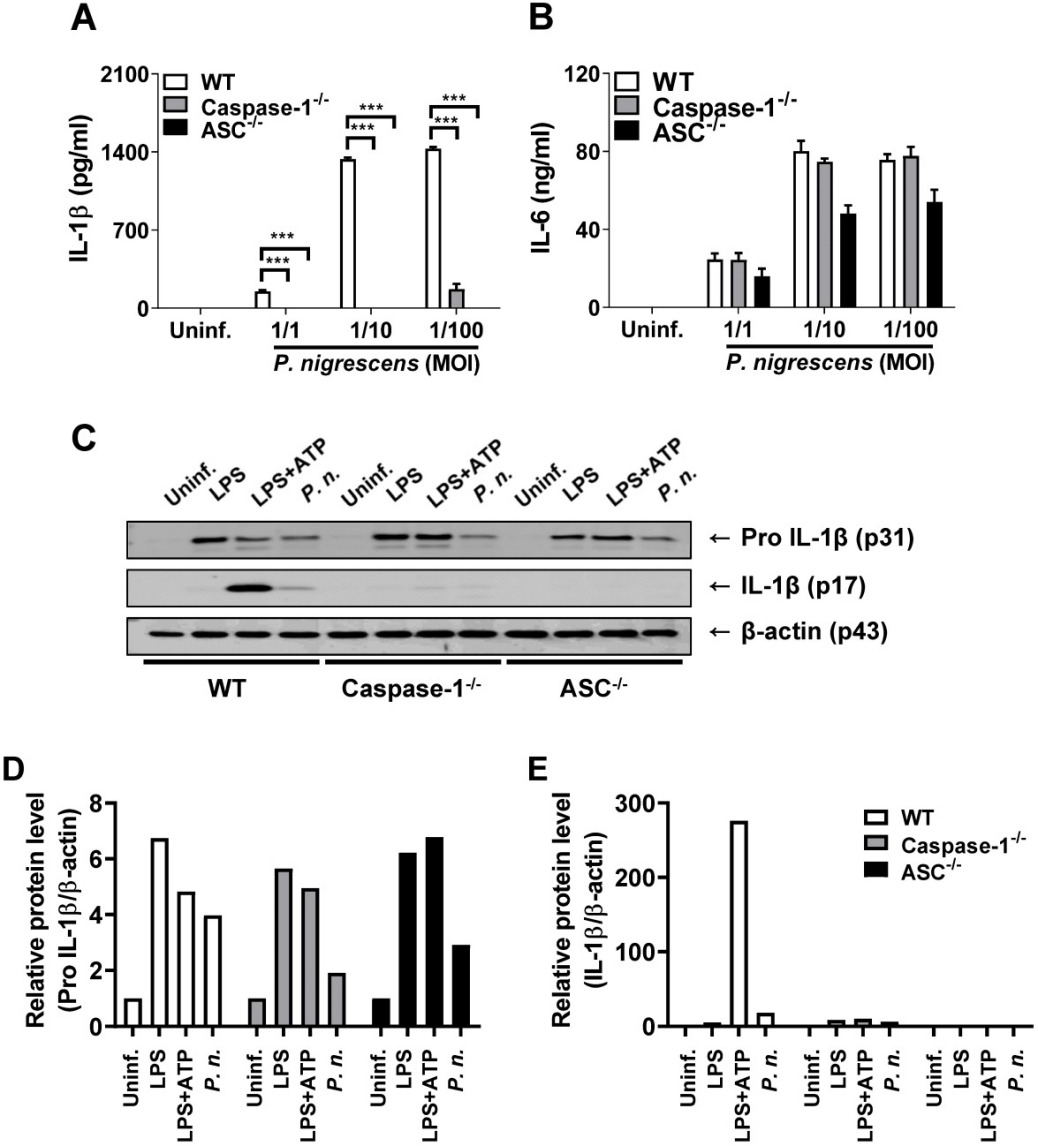
** Caspase-1 and ASC are required for *P. nigrescens*-induced processing of IL-1β in BMDCs.** BMDCs from WT, Caspase-1-, and ASC-deficient mice were infected with *P. nigrescens* (A and B) at an MOI of 100 for 6 h or (C) at the indicated MOI for 24 h. The production of (A) IL-1β and (B) IL-6 was determined by ELISA and (C) the cleaved and immature forms of IL-1β were analyzed by Western blotting. (D and E) The intensity of each band was quantified with ImageJ software and normalized to β-actin. The results are from one experiment representative of three independent experiments and expressed as mean ± SD. *P < 0.05, **P < 0.01, and ***P < 0.001.

Proteolytic cleavage of the inactive precursor IL-1β by several proteases including caspase-1 is required to generate its biologically active form 19. To determine whether caspase-1 is required for IL-1β processing in *P. nigrescens*-infected BMDCs, BMDCs from WT, caspase-1-, and ASC-deficient mice were infected with *P. nigrescens* and IL-1β processing was examined. The production of IL-1β but not IL-6 in BMDCs from capase-1- and ASC-deficient mice was impaired (Fig. 3A and B). Immature pro-IL-1β production was comparable in *P. nigrescens*-infected BMDCs from WT, caspase-1-, and ASC-deficient mice (Fig. 3C). However, cleavage of pro-IL-1β to mature IL-1β was impaired in BMDCs from caspase-1- and ASC-deficient mice (Fig. 3C). These results indicate that caspase-1 and ASC are required for processing of IL-1β in *P. nigrescens*-infected BMDCs.

### The NLRP3 inflammasome contributes to caspase-1 activation and IL-1β production in *P. nigrescens*-infected BMDCs

**Figure 4:**
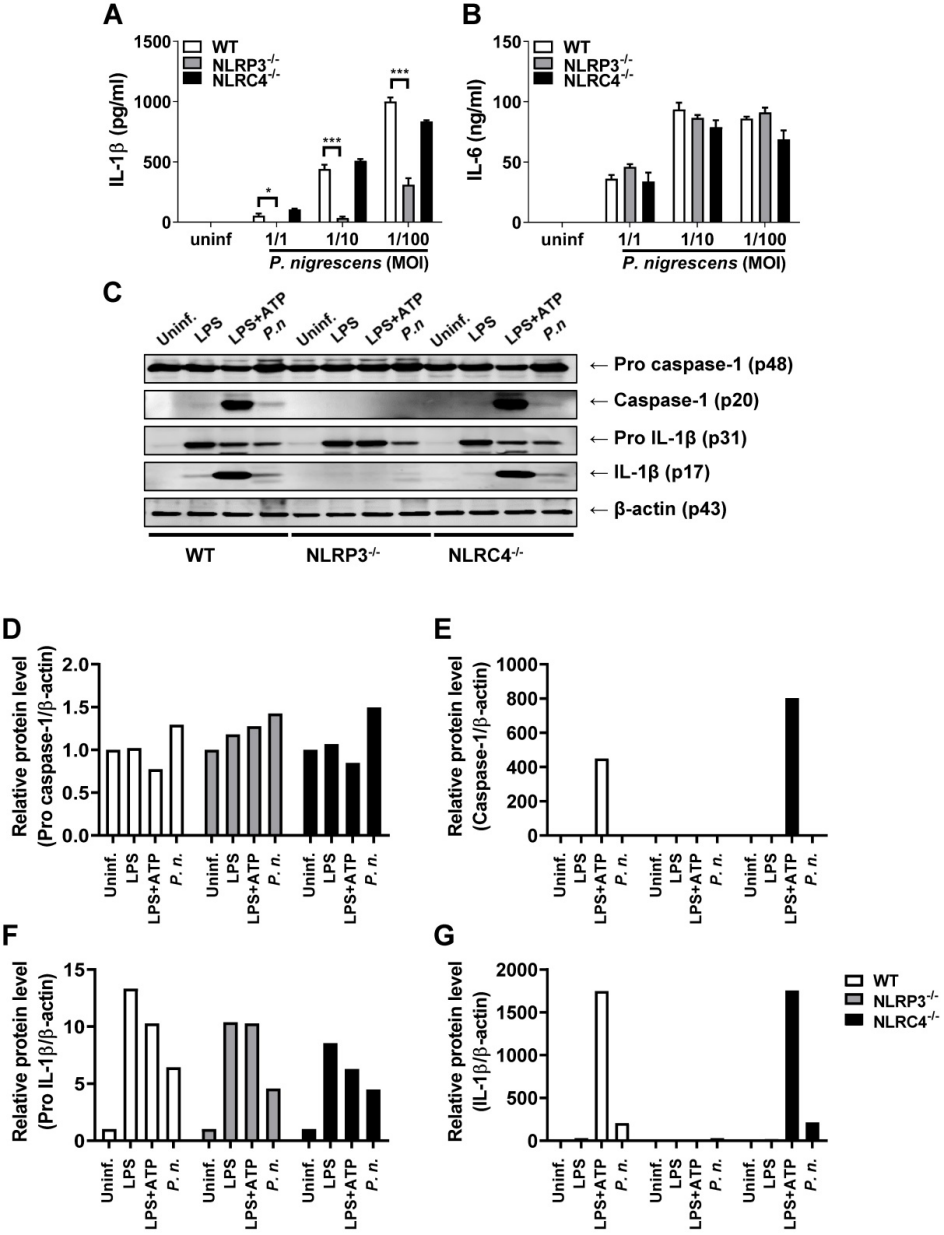
** Deficiency of NLRP3 but not NLRC4 impairs caspase-1 activation and IL-1β processing in *P. nigrescens*-infected BMDCs.** BMDCs from WT, NLRP3-, and NLRC4-deficient mice were infected with *P. nigrescens* (A and B) at the indicated MOI for 24 h or (C) at an MOI of 100 for 6 h. Production of (A) IL-1β and (B) IL-6 was determined by ELISA and (C) the cleaved forms of caspase-1 and IL-1β and their immature forms were analyzed by Western blotting. (D-G) The intensity of each band was quantified with ImageJ software and normalized to β-actin. The results are from one experiment representative of three independent experiments and expressed as mean ± SD. *P < 0.05, **P < 0.01, and ***P < 0.001.

**Figure 5:**
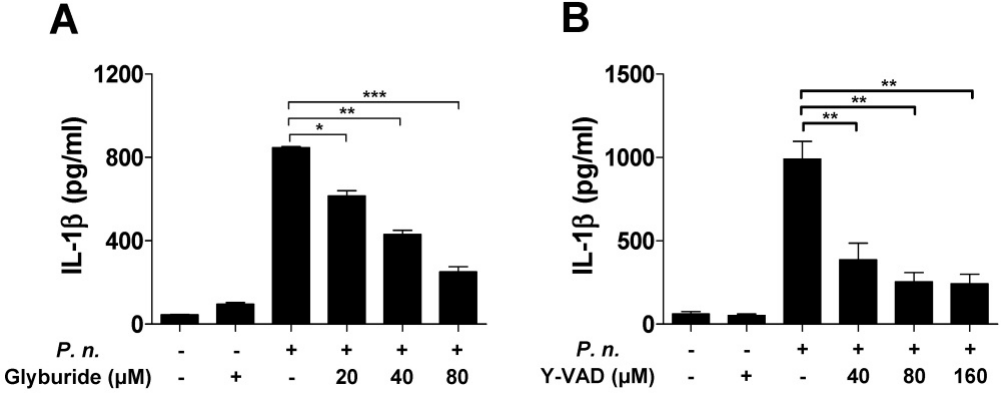
** IL-1β secretion by *P. nigrescens*-infected BMDCs requires activation of caspase-1 and the NLRP3 inflammasome.** BMDCs were infected with *P. nigrescens* (MOI 10) for 24 h with or without pretreatment with (A) Glyburide (20 to 80 µM) and (B) Ac-YVAD-CMK (40 to 160 µM) and the level of IL-1β in culture supernatants was analyzed by ELISA. The results are from one experiment representative of three independent experiments and expressed as mean ± SD. *P < 0.05, **P < 0.01, and ***P < 0.001.

Several inflammasomes can trigger caspase-1 activation and IL-1β maturation 12,16,20. To identify the inflammasome that induces caspase-1 activation in BMDCs infected with *P. nigrescens*, BMDCs from WT, NLRP3-, and NLRC4-deficient mice were infected with *P. nigrescens*. IL-1β secretion following *P. nigrescens* infection was impaired in BMDCs from NLRP3-deficient mice, but not in BMDCs from NLRC4-deficient mice (Fig. 4A). IL-6 secretion following *P. nigrescens* infection was comparable in BMCS from WT, NLRP3-, and NLRC4-deficient mice (Fig. 4B). Caspase-1 activation and IL-1β maturation were impaired in BMDCs from NLRP3-deficient mice but not in BMDCs from NLRC4-deficient mice in response to *P. nigrescens* infection (Fig. 4C). In addition, pretreatment with Glyburide (an NLRP3 inhibitor) or Ac-YVAD-CMK (a caspase-1 inhibitor) impaired IL-1β production in *P. nigrescens*-infected BMDCs (Fig. 5A and B). These results indicate that the NLRP3 inflammasome is required for maturation of IL-1β in* P. nigrescens*-infected BMDCs.

### ROS generation, P2X_7_R, and lysosomal destabilization are involved in IL-1β production in *P. nigrescens*-infected BMDCs

**Figure 6:**
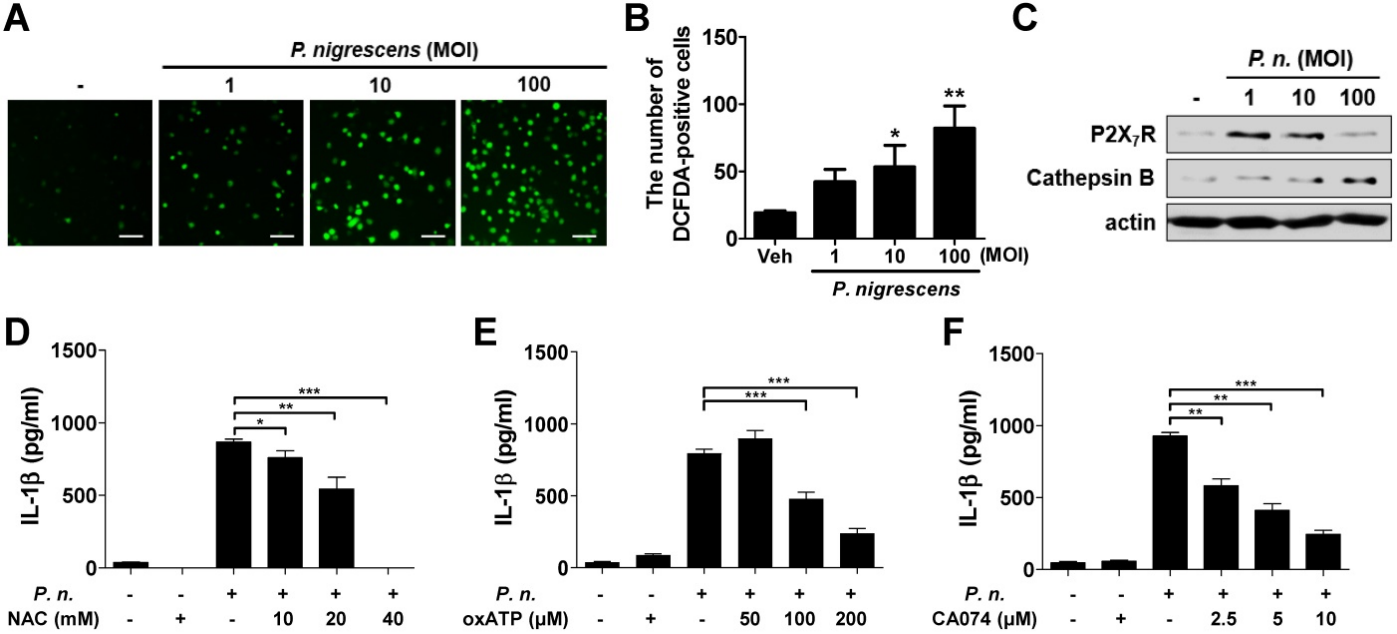
** Reactive oxygen species production, extracellular ATP, and lysosomal destabilization are involved in IL-1β production in *P. nigrescens*-infected BMDCs**. BMDCs were infected with indicated dose of *P. nigrescens* for 24 h (A-C). ROS production was determined by DCFDA staining (A and B) and expression of P2X_7_R and cathepsin B was by Western blot (C). For an inhibitor assay, BMDCs were infected with the bacteria (MOI 10) for 24 h with or without pretreatment with (D) NAC (10 to 40 mM), (E) oxATP (50 to 200 µM), or (F) CA-074 Me (2.5 to 10 µM) for 2 h. Production of IL-1β was determined by ELISA. The results are from one experiment representative of three independent experiments and expressed as mean ± SD. *P < 0.05, **P < 0.01, and ***P < 0.001.

ROS generation, P2X_7_ receptor (P2X_7_R) activation, and cathepsin B release from lysosomes are known to be involved in NLRP3 inflammasome activation and IL-1β production 1,20-22. We first evaluated whether *P. nigrescens* promotes ROS production and expression of P2X_7_R and cathepsin B in BMDCs. Results showed that *P. nigrescens* increases the number of DCFDA-positive cells in a dose-dependent manner (Fig. 6A). Expression of P2X_7_R and cathepsin B was also increased by *P. nigrescens* (Fig. 6B and C). To examine the involvement of these factors in IL-1β production by *P. nigrescens*-infected BMDCs, BMDCs were pretreated with N-acetyl cysteine (NAC, a ROS scavenger), oxATP (a P2X_7_R inhibitor), or CA-074 Me (a cathepsin B inhibitor). Pretreatment with NAC, oxATP, or CA-074 Me impaired IL-1β production in *P. nigrescens*-infected BMDCs (Fig. 6D-F). However, IL-6 production was not impaired by pretreatment with NAC, oxATP, or CA-074 Me (data not shown). An MTT assay revealed that treatment with these compounds was not cytotoxic (data not shown). These results indicate that ROS generation, P2X_7_R activation, and cathepsin B are involved in IL-1β production in *P. nigrescens*-infected BMDCs.

## Discussion

Previous clinical reports showed that the amount of IL-1β is much higher in periodontitis pockets and in inflamed gingival tissue than in healthy tissue and that an increase of IL-1β activity in gingival crevicular fluid is correlated with clinical signs of periodontitis such as gingival index and proving depth 23. As increased *P. nigrescens* colonization seems to be associated with periodontitis 8, we can speculate that the production of IL-1β in gingival tissue with *P. nigrescens* infection may play a role in the pathogenesis of periodontitis. In this study, we identified the host factors that induce IL-1β processing and secretion following *P. nigrescens* infection using DCs, which are known to play a pivotal role in the progression of periodontitis 6. Based on our results using TLR2- and TLR4-deficient dendritic cells, TLR2 but not TLR4 was required for transcriptional expression of pro-IL-1β in response to *P. nigrescens* infection. In addition, an increase of NLRP3 expression, which is an essential prerequisite to activation of the NLRP3 inflammasome, required TLR2 but not TLR4 in dendritic cells. Previously, Kikkert et al. examined the interaction of *Porphyromonas gingivalis*, *Actinobacillus actinomycetemcomitans*, *Tannerella forsythensis*, *Prevotella intermedia*, *P. nigrescens*, *Fusobacterium nucleatum,* and *Veillonella parvula* with TLR2 and TLR4 using human embryonic kidney cells stably transfected with TLR2 or TLR4 18. In accordance with our results, all tested bacteria including *P. nigrescens* stimulated TLR2 but only *A. actinomycetemcomitans* and *V. parvula* stimulated TLR4 18.

Pro-IL-1β requires enzymatic cleavage to be biologically active 12. We showed that caspase-1 was required to process pro-IL-1β into mature IL-1β in *P. nigrescens*-infected DCs. Several inflammasomes including the NLRP3 and NLRC4 inflammasomes are known to activate caspase-1; we showed that *P. nigrescens* activated the NLRP3 inflammasome and processed pro-IL-1β into mature IL-1β in DCs. In contrast, the NLRC4 inflammasome appears to be dispensable for caspase-1 activation and IL-1β production in DCs in response to *P. nigrescens* infection. The NLRC4 inflammasome, which responds to bacterial flagellin and the needle and rod proteins of the type III secretion system (T3SS) with the assistance of NLR family apoptosis inhibitory proteins (NAIPs), results in activation of caspase-1 and processing of IL-1β 24. As *Prevotella* species are known to be non-motile and utilize the type IX secretion system (T9SS), which was originally identified in *P. gingivalis* 25, NLRC4 signaling may not be essential for *P. nigrescens*-induced IL-1β secretion and caspase-1 activation in DCs.

Several cellular mechanisms have been proposed to contribute to activation of the NLRP3 inflammasome, including generation of ROS, K^+^ efflux, and cathepsin B release from damaged lysosomes 20,26. Our results show that inhibition of ROS generation, P2X_7_R receptor activation, and cathepsin B impaired the production of IL-1β in dendritic cells in response to *P. nigrescens*. The clinical report that demonstrated increased ROS in inflamed periodontal tissue 27 implicated ROS in IL-1β-related pathogenesis in periodontitis following *P. nigrescens* infection. Extracellular ATP is known to lead to activation of P2X_7_R, resulting in K^+^ efflux which in turn activates the NLRP3 inflammasome 28-30, and the release of lysosomal cathepsin B has been shown to activate the NLRP3 inflammasome 31. In accordance with our results, a recent study demonstrated that a common oral pathogen, *P. gingivalis,* induces NLRP3 inflammasome in response to ATP, K^+^ efflux, and cathepsin B release in macrophages 1. In addition, the previous report suggested that the amount of cathepsin B in gingival crevicular fluid is closely associated with disease severity and that cathepsin B plays an important role in the pathogenesis of periodontitis 32.

In conclusion, our results show that the production of IL-1β in dendritic cells following *P. nigrescens* infection requires TLR2 and the NLRP3 inflammasome with generation of ROS, K^+^ efflux, and cathepsin B release. These molecules provide potential targets to control IL-1β-related periodontitis in *P. nigrescens*-infected patients.
